# miR-500a-3p promotes cancer stem cells properties via STAT3 pathway in human hepatocellular carcinoma

**DOI:** 10.1186/s13046-017-0568-3

**Published:** 2017-07-27

**Authors:** Chunlin Jiang, Jianting Long, Baoxian Liu, Ming Xu, Wei Wang, Xiaoyan Xie, Xiaolin Wang, Ming Kuang

**Affiliations:** 1 Department of Medical Ultrasonics, Division of Interventional Ultrasound, Institute of Diagnostic and Interventional Ultrasound, The First Affiliated Hospital, Sun Yat-sen University, Guangzhou, 510080 China; 2 Department of Medicinal Oncology, The First Affiliated Hospital, Sun Yat-sen University, Guangzhou, 510080 China; 3 Guangdong Institute of Gastroenterology, Guangdong Provincial Key Laboratory of Colorectal and Pelvic Floor Diseases, The Sixth Affiliated Hospital, Sun Yat-sen University, Guangzhou, 510080 China; 4 Department of liver surgery, The First Affiliated Hospital, Sun Yat-sen University, Guangzhou, 510080 China

**Keywords:** miR-500a-3p, Cancer stem cell, STAT3 signaling pathway, Hepatocellular carcinoma

## Abstract

**Background:**

miR-500a-3p has been demonstrated to be involved in the development, progression and metastasis in several human cancers. Constitutive activation of JAK/STAT3 signaling pathway has been reported to play an important role in the development and progression of hepatocellular carcinoma (HCC).The purpose of this study was to determine the biological roles and clinical significance of miR-500a-3p in HCC and to identify whether miR-500a-3p has an effect on the activity of JAK/STAT3 signaling in HCC.

**Methods:**

miR-500a-3p expression was examined by real-time PCR in 8 paired HCC tissues and individual 120 HCC tissues respectively. Statistical analysis was performed to explore the clinical correlation between miR-500a-3p expression and clinicopathological features and overall and relapse-free survival in HCC patients. In vitro and in vivo assays were performed to investigate the biological roles of miR-500a-3p in HCC. The bioinformatics analysis, real-time PCR, western blot and luciferase reporter assay were performed to discern and examine the relationship between miR-500a-3p and its potential targets. Clinical correlation of miR-500a-3p with its targets was examined in HCC tissues.

**Results:**

miR-500a-3p is dramatically elevated in HCC tissues and cells and high expression of miR-500a-3p correlates with poor overall and relapse-free survival in HCC patients. Upregulating miR-500a-3p enhances, while silencing miR-500a-3p suppresses, the spheroid formation ability, fraction of side population and expression of cancer stem cell factors in vitro and tumorigenicity in vivo in HCC cells. Our findings further reveal miR-500a-3p promotes the cancer stem cell characteristics via targeting multiple negative regulators of JAK/STAT3 signaling pathway, including SOCS2, SOCS4 and PTPN11, leading to constitutive activation of STAT3 signaling. Moreover, the inhibitory effects of anti-miR-500a-3p on cancer stem cell phenotypes and activity of STAT3 signaling were reversed by silencing SOCS2, SOCS4 and PTPN11 in miR-500a-3p-downexpressing cells, respectively. Clinical correlation of miR-500a-3p with the targets was examined in human HCC tissues.

**Conclusion:**

our results uncover a novel mechanism by which miR-500a-3p promotes the stemness maintenance of cancer stem cell in HCC, suggesting that silencing miR-500a-3p may serve as a new therapeutic strategy in the treatment of hepatocellular carcinoma.

**Electronic supplementary material:**

The online version of this article (doi:10.1186/s13046-017-0568-3) contains supplementary material, which is available to authorized users.

## Background

Hepatocellular carcinoma (HCC) is one of the most malignant cancers with dismal prognosis due to its high rate of recurrence, which can be as high as 70% [1]. Liver transplantation or surgical resection is the first-line treatment for HCC, but the majority of patients with HCC are inoperable due to the advanced stages. For the most advanced HCC, the long-term prognosis of HCC remains unsatisfactory even following conventional treatments, including transarterial chemoembolization or a systemic embolization, chemotherapy and radiofrequency ablation, due to the chemoresistant nature of the cancer cells [2, 3]. The primary cause responsible for the failure of traditional treatments is the existence of cancer stem cells (CSCs). CSCs are a minority cell population within tumors and characterized by unlimited proliferation and the abilities of self-renewal and differentiation into the heterogeneous lineages of cancer cells. These features of CSCs contribute to a hierarchical organization of cancer cells [4, 5]. Therefore, further investigation into the mechanism that regulates the maintenance of CSCs may help to develop novel therapies aimed at eradicating the CSC population to achieve long-term remission and improve the survival rate in HCC patients.

Several lines of evidence indicated that the Janus kinase/signal transducer and activator of transcription (JAK/STAT) pathway plays an important role in the regulation of CSC self-renewal and is constitutively activated in a number of human cancers [6]. It’s worth noting that some cancer stem cell factors, such as Nanog homeobox (NANOG), are well-known targets of STAT3 signaling pathway, further elucidating the regulatory role of STAT3 signaling in the maintenance of CSCs [7]. From a molecular perspective, JAK/STAT3 signaling is activated by binding to different types of ligands,such as interleukin-6 (IL-6), interferon (IFN), and IL-10, which induces receptor dimerization, activating the associated JAKs. The activated JAKs engage with cytokine receptors and then phosphorylate tyrosine residues on the receptor, which further recruits and phosphorylate STATs. The phosphorylated STATs are released from the receptors and form homo- or hetero- dimers and translocate to the nucleus where they drive the transcription of target genes [8]. Numerous studies have revealed JAK/STAT signaling pathway was implicated in several aspects of tumorigenesis, including proliferation, apoptosis, angiogenesis, and metastasis [9]. These evidence demonstrated that JAK/STAT3 signaling pathway plays crucial roles in the tumorigenesis and progression of cancer.

Suppressor of cytokine signaling (SOCS) and protein tyrosine phosphatase are well-documented negative regulators of JAK/STAT signaling pathway, which tightly control the activity of JAK/STAT signaling [10, 11]. SOCS family proteins form a classic negative feedback system via inhibiting JAK kinase activity and STATs binding to cytokine receptors, and facilitating proteasomal degradation of JAK [12]. Protein tyrosine phosphatases, which are a group of enzymes that catalyse the removal of a phosphate group from phosphorylated tyrosine residues, play a regulatory role in various cell signal transduction pathways that are critical for a diversity of cell functions, including transcription regulation, metabolic control and cell migration [13]. Accumulating studies indicated that loss or downregulation of SOCS proteins or tyrosine phosphatase contributed to the development and progression of cancer. Maria reported that the absence of SOCS molecules promoted the propagation of the JAK/STAT3 signaling. Exogenous expression of SOCS1, SOCS3, and SOCS5 in the highly aggressive anaplastic thyroid cancer cells abolished the activation of STAT3 signaling pathway activation resulting in alteration in the balance of proapoptotic and antiapoptotic molecules and sensitization to chemotherapeutic drugs in vitro [14]. Furthermore, mutation or loss of PTPN11, a tyrosine phosphatase, have also been detected in number of cancer, including breast cancer, gastric cancer, acute myeloid leukemia, lung cancer, colorectal cancer [15, 16]. These studies further elucidated the anti-tumor roles of these negative regulators of JAK/STAT3 signaling pathway in human cancer.

MiRNAs are short, noncoding RNAs and regulate the expression of target genes in the posttranscriptional level via binding to the 3′ untranslated region (3’UTR) of downstream target genes, resulting in the mRNA degradation and/or translational inhibition [17]. MiRNAs are mechanistically involved in a variety of biological processes, including cell apoptosis, proliferation and differentiation [17, 18]. A number of studies reported that dysregulation of miRNAs contributed to the tumorigenesis and progression of various kinds of cancers by regulating CSCs, including hepatocellular carcinoma [19–22]. miR-500a-3p, as one of the original miRNAs discovered, has been reported to be implicated in the chemotherapeutic resistance, invasion and migration via SFRP2, GSK-3β and LY6K in different types of cancers [23, 24]. However, the biological roles of miR-500a-3p and the underlying molecular mechanisms responsible for HCC initiation and progression have not been reported. In this study, we found that miR-500a-3p is evevated in HCC tissues and overexpression of miR-500a-3p correlates with poor survival in HCC patients. Furthermore, upregulating miR-500a-3p promotes, while silencing miR-500a-3p inhibits the cancer stem cell properties in vitro and tumorigenicity in vivo in HCC cells. Our results further demonstrated that miR-500a-3p promotes the CSC-like phenotypes via targeting negative regulators SOCS2, SOCS4 and PTPN11 of STAT3 signaling pathway, leading to the activation of STAT3 signaling. Importantly, the stimulatory effects of miR-500a-3p on stem cell properties were antagonized by STAT3 inhibitors Stattic and S3I-201 in miR-500a-3p-overexpressing cells. These findings reveal critical roles for miR-500a-3p in STAT3 signaling and tumorigenicity of hepatocellular carcinoma, and may be identified as a novel therapeutic target against hepatocellular carcinoma.

## Methods

### Cell lines and cell culture

The human hepatocellular carcinoma cell lines 97H, Hep G2, QGY-7703, Hep 3B, PLC, Huh 7 and SMMC7721 were obtained from American Type Culture Collection (ATCC, USA), and all human hepatocellular carcinoma cell lines were cultured as ATCC protocol described. Human liver immortal cell line L02 was purchased from Biomics Biotechnologies (Nantong, China). The cell lines were maintained in RPMI 1640 (Invitrogen, USA) supplemented with 10% FBS (HyClone, USA) and 100 units/ml penicillin plus 100 μg/ml streptomycin. Cells were grown under a humidified atmosphere of 5% CO2 at 37 °C.

### Patients and tumor tissues

Eight paired hepatocellular carcinoma tissues with the matched adjacent normal tissues and 120 separate hepatocellular carcinoma tissues were obtained during surgery at the First Affiliated Hospital of Sun Yat-sen University (Guangzhou, China) between 2006 and 2010. Patients were diagnosed based on clinical and pathological evidence, and the specimens were immediately snap-frozen and stored in liquid nitrogen tanks. Briefly, total RNA from HCC tissues and the matched adjacent normal tissues was extracted using RNA Isolation Kit (Cat No. 338904#, Qiagen, USA) according to the manufacturer’s instructions. mRNA was reverse transcribed of total mRNA using the RevertAid First Strand cDNA Synthesis Kit (Cat No. K1622, Thermo, USA) according to the manufacturer’s protocol. Complementary DNA (cDNA) was amplified and quantified on CFX96 system (BIO-RAD, USA) using iQ SYBR Green (BIO-RAD, USA). U6 was used as endogenous controls. The expression of miRNA was defined based on Ct, and relative expression levels were calculated as 2^-(Ct^_miR-500a-3p_^- Ct^_U6_^)^ after normalization with reference to the quantification of U6 small nuclear RNA expression For the use of these clinical materials for research purposes, prior patients’ consents and approval from the Institutional Research Ethics Committee were obtained. The median of miR-500a-3p expression in HCC tissues was used to stratify high and low expression of miR-500a-3p.

### Plasmids, transfection and generation of stable cell lines

The miR-500a-3p expression plasmid was generated by cloning the genomic pre–miR-500a-3p gene, with a 300-bp sequence on each flanking side, into retroviral transfer plasmid pMSCV-puro (Clontech Laboratories Inc.) to generate plasmid pMSCV- miR-500a-3p. pMSCV- miR-500a-3p was cotransfected with the pIK packaging plasmid in 293FT cells using the standard calcium phosphate transfection method, as previously described [25]. Thirty-six hours after the co-transfection, supernatants (MOI: 20) were collected and incubated with cells to be infected for 24 h in the presence of polybrene (2.5 μg/ml). After infection, puromycin (1.5 μg/ml) was used to select stably transduced cells over a 10-day period. The 3’UTR of SOCS2, SOCS4 and PTPN11 were amplified and cloned downstream to the luciferase gene in a modified pGL3 control vector (Promega, USA), and the list of primers used in clone reactions was presented in Additional file 1: Table S1. Anti-miR-500a-3p, small interfering RNA (siRNA) for SOCS2, SOCS4 and PTPN11 knockdown and respective control RNA (50 nM) were synthesized and purified by RiboBio. The sequence of anti-miR-500a-3p is cagaauccuugcccaggugcau. Transfection of miRNAs, siRNAs, and plasmids was performed using Lipofectamine 3000 (Life Technologies) according to the manufacturer’s instructions.

### RNA extraction, reverse transcription, and real-time RT-PCR

Total RNA from tissues or cells was extracted using RNA Isolation Kit (Cat No. 338904#, Qiagen, USA) according to the manufacturer’s instructions. Messenger RNA (mRNA) and miRNA were reverse transcribed of total mRNA using the RevertAid First Strand cDNA Synthesis Kit (Cat No. K1622, Thermo, USA) according to the manufacturer’s protocol. Complementary DNA (cDNA) was amplified and quantified on CFX96 system (BIO-RAD, USA) using iQ SYBR Green (BIO-RAD, USA). Detection of mRNA was performed as described previously [26]. The sequences of the primers were listed in Additional file 2: Table S2. U6 or glyceraldehyde-3-phosphate dehydrogenase (GAPDH) was used as endogenous controls. The expression of miRNA was defined based on Ct, and relative expression levels were calculated as 2^-(Ct^_miR-500a-3p_^- Ct^_U6_^)^ after normalization with reference to the quantification of U6 small nuclear RNA expression and expression levels of mRNA were calculated after normalization with reference to the quantification of glyceraldehyde-3-phosphate dehydrogenase (GAPDH) RNA expression according to the previous study [27].

### Western blot

Western blot was performed according to a standard method, as described previously [28]. The following primary antibodies were used: anti–SOCS2, anti–SOCS4, anti–SOCS6, anti–PTPN4, anti–PTPN11, anti–STAT3 and a-tubulin (Cell Signaling Technology); p84 was purchased from Abcam (Cambridge, USA). Nuclear extracts were prepared using the Nuclear Extraction Kit (Active Motif), according to the manufacturer’s instructions. All protein expression levels in this manuscript were quantified by densitometry using Quantity One Software, and normalized to the corresponding expression levels of α-tubulin respectively. The sample 1 was used as a standard.

### Luciferase reporter assay

Cells were seeded in triplicate in 24-well plates and allowed to settle for 24 h. The 3’UTR of SOCS2, SOCS4 and PTPN11 were amplified and cloned downstream to the luciferase gene in a modified pGL3 control vector (Promega, USA), and the plasmid phRL-tk was used as the internal control for transfection efficiency and cytotoxicity of test chemicals (Promega). The 20 or 50 μg 3’UTR of SOCS2, SOCS4 and PTPN11 plasmids and 1μg pRL-TK Renilla plasmid were transfected into the cells using Lipofectamine 3000 Reagent (Life Technologies). Forty-eight hours after transfection, Dual- Luciferase Reporter Assay (Promega) was performed according to the manufacturer’s instructions, as previously described [29].

### Tumor xenografts

All experimental procedures were approved by the Institutional Animal Care and Use Committee of Sun Yat-sen University. The 6-week-old BALB/c-nu mice were randomly divided into four groups (*n* = 6 per group). Cells (1 × 10^5^, 1 × 10^4^ and 1 × 10^3^) were inoculated subcutaneously together with Matrigel (final concentration of 25%) into the inguinal folds of the nude mice respectively. Tumor volume was determined using an external caliper and calculated using the eq. (L × W^2^)/2. The mice were sacrificed 35 days after inoculation and the tumors were excised and subjected to pathological examination.

### Side population analysis

The cell suspensions were labeled with Hoechst 33,342 (Molecular probes – #H-3570) dye for side population analysis as per standard protocol [30]. Briefly, cells were resuspended at 1× pre-warmed OptiMEM (Gibco, USA) containing 2% FBS (Gibco, USA) at a density of 10^6^/mL. Hoechst 33,342 dye was added at a final concentration of 5 lg/mL in the presence or absence of verapamil (50 lmol/L; Sigma) and the cells were incubated at 37 °C for 90 min with intermittent shaking. At the end of the incubation, the cells were washed with OptiMem containing 2% FBS and centrifuged down at 4 °C, and resuspended in ice-cold OptiMem containing 2% FBS and 10 mmol/L HEPES. Propidium iodide (Sigma, USA) at a final concentration of 2 lg/mL was added to the cells to gate viable cells. The cells were filtered through a 40-lm cell strainer to obtain single cell suspension before sorting. Analysis and sorting was done on a FACS AriaI (Becton Dickinson). The Hoechst 33,342 dye was excited at 355 nm and its dual-wavelength emission at blue and red region was plotted to get the SP scatter.

### miRNP immunoprecipitation

Cells were co-transfected with HA-Ago1 together with 100 nM miR-500a-3p, followed by HA-Ago1 immunoprecipitation using HA-antibody. Real-time PCR analysis of the IP material was used to test the association of the mRNA of DKK1 with the RISC complex. The specific processes were performed as described previously [31].

### Statistical analysis

All statistical analyses were carried out using the SPSS 16.0 statistical software package. Student’s t-test was used to determine statistical differences between two groups. One-way ANOVA was used to determine statistical differences between multiple testing. The chi-square test was used to analyze the relationship between miR-500a-3p expression and clinicopathological characteristics. Survival curves were plotted using the Kaplan Meier method and compared by log-rank test. *P* < 0.05 was considered significant. All the experiments were repeated three times. In all cases, *P* < 0.05 was considered significant. All the experiments were repeated at least three times.

## Results

### miR-500a-3p is upregulated in hepatocellular carcinoma and associated with poor survival

**Fig. 1:**
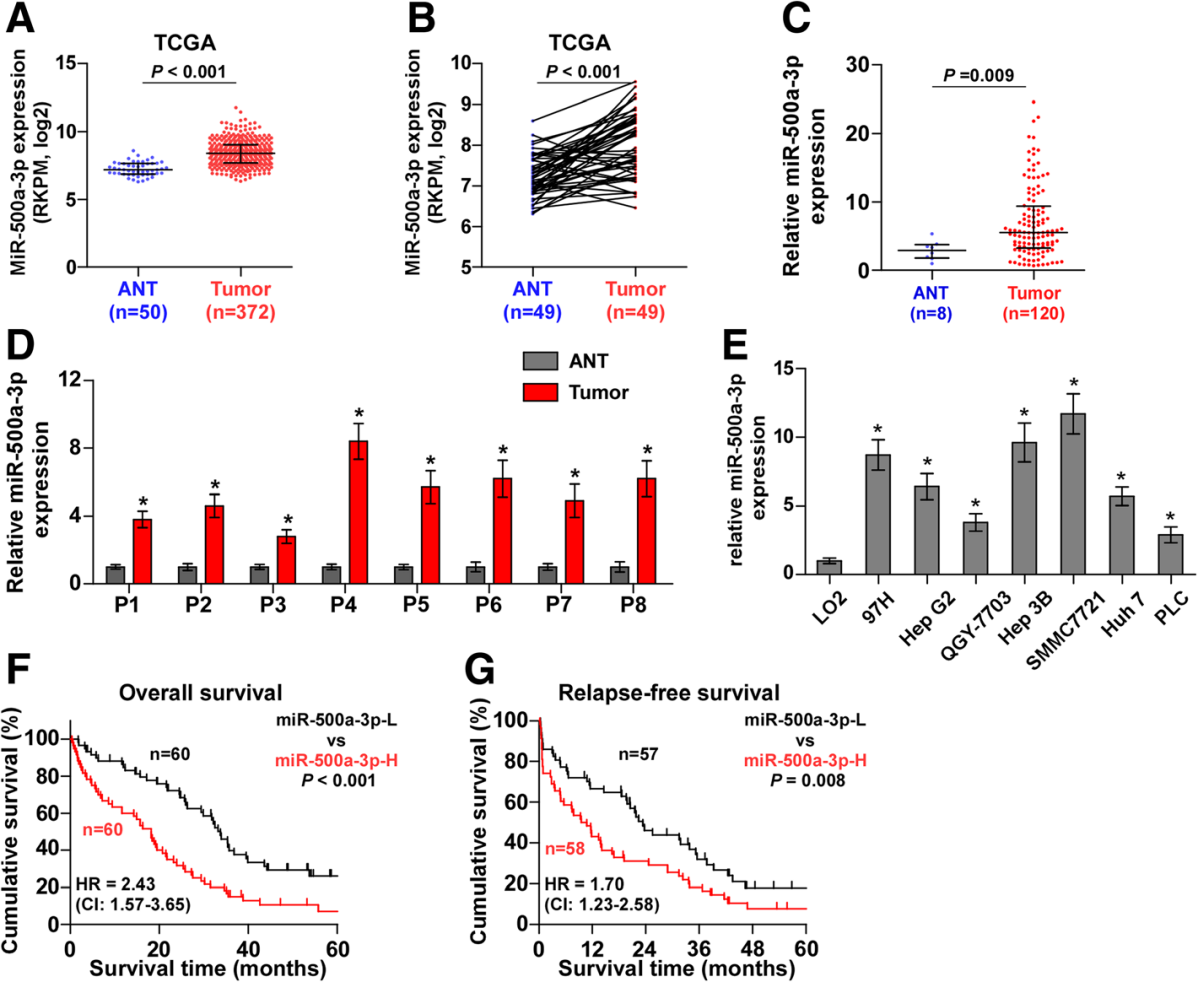
miR-500a-3p is upregulated in hepatocellular carcinoma and correlates with poor prognosis. **a** miR-500a-3p expression level in normal hepatic tissues and hepatocellular carcinoma tissues in the miRNA sequencing dataset of the TCGA hepatocellular carcinoma (Normal, *n* = 50; Hepatocellular carcinoma, *n* = 372). *P* < 0.001. **b** miR-500a-3p expression level in 49 paired primary hepatocellular carcinoma tissues compared with matched adjacent normal tissues. Transcript levels were normalized to U6 expression. *P* < 0.001. **c** Real-time PCR analysis of miR-500a-3p expression in 120 hepatocellular carcinoma tissues compared to the 8 matched adjacent normal hepatic tissues. Transcript levels were normalized to U6 expression. *P* < 0.05. **d** miR-500a-3p expression level in 8 paired primary hepatocellular carcinoma tissues compared with matched adjacent normal tissues. Transcript levels were normalized to U6 expression. **e** miR-500a-3p expression level in one normal hepatic cell and seven hepatocellular carcinoma cells. **f** Kaplan–Meier analysis of overall and relapse-free survival curves of patients with hepatocellular carcinoma in high miR-500a-3p expression (*n* = 60) and low miR-500a-3p expression (*n* = 60). *P* < 0.001, log-rank test. The data shown in scatter plot and bar graph were determined by the median with interquartile range and median with standard deviation.**g** Kaplan–Meier analysis of overall survival curves of patients with hepatocellular carcinoma from TCGA in high miR-500a-3p expression (*n* = 174) and low miR-500a-3p expression (*n* = 173). *P* < 0.001

To screen the potential miRNA in HCC, the hepatocellular carcinoma dataset from The Cancer Genome Atlas (TCGA) and ArrayExpress was analyzed and revealed that miR-500a-3p was upregulated in HCC tissues compared with adjacent normal tissues (Fig. 1a and b and Additional file 3: Figure S1A and 1B). We further measured the miR-500a-3p expression in our own HCC tissue samples (Additional file 4: Table S3). Consistently, miR-500a-3p expression was markedly elevated in HCC tissues compared with that in the 8 matched tumor-adjacent normal hepatic tissues (ANT) (Fig. 1c). The expression of miR-500a-3p in eight paired HCC tissues was increased compared with the matched adjacent normal tissues (Fig. 1d). We further examined the expression level of miR-500a-3p in HCC cells and found that compared to human liver immortal cell line L02, the expression of miR-500a-3p was differentially elevated in seven hepatocellular carcinoma cells (Fig. 1e). We further investigated the clinicopathological characteristics and prognostic significance of miR-500a-3p expression in HCC. As shown in Additional file 5: Table S4, miR-500a-3p expression was positively associated with AFP expression level (*P* = 0.026), tumor size (*P* = 0.041), venous invasion (*P* = 0.029), distant metastasis (*P* = 0.006) and clinical stage (*P* = 0.010) in HCC patients. Kaplan–Meier survival analysis revealed that patients with high miR-500a-3p expression correlated with poor overall and relapse-free survival (Fig. 1f and g), which was consistent with the TCGA analysis from HCC dataset (Additional file 3: Figure S1C and 1D). Thus, our fingding demonstrated that miR-500a-3p expression is elevated in HCC tissues and cells and correlates with poor survival in HCC patients.

### miR-500a-3p targets negative regulator of STAT3 signaling pathway

**Fig. 2:**
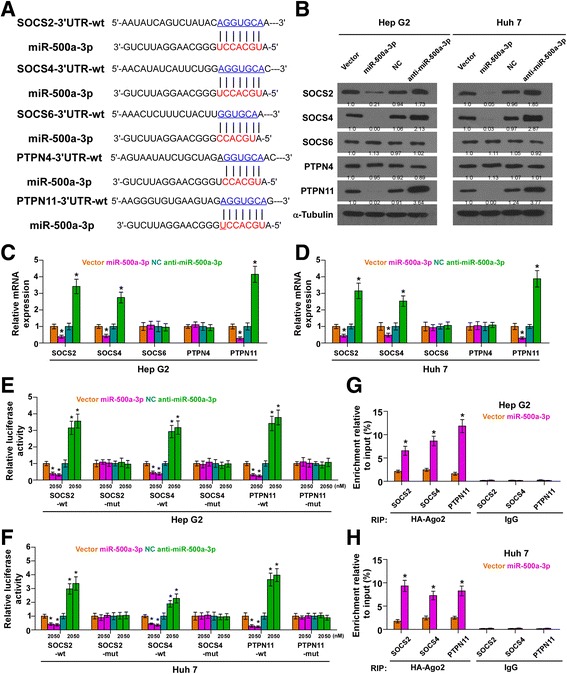
miR-500a-3p targets multiple negative regulators of STAT3 signaling. **a** Predicted miR-500a-3p target sequence in 3′UTRs of SOCS2, SOCS4, SOCS6, PTPN4 and PTPN11 **b** Western blotting of SOCS2, SOCS4, SOCS6, PTPN4 and PTPN11 expression after transfected with miR-500a-3p mimics or miR-500a-3p inhibitor compared to control . α-Tubulin served as the loading control. **c** and **d** Real-time PCR analysis of SOCS2, SOCS4, SOCS6, PTPN4 and PTPN11 expression in Hep G2 and Huh 7 cells transduced with miR-500a-3p mimics or transfected with miR-500a-3p inhibitor compared to control. Transcript levels were normalized by GAPDH expression. Error bars represent the mean ± s.d. of three independent experiments. *P < 0.05. **e** and **f** Luciferase assay of cells transfected with pmirGLO-3′UTR reporter in the indicated Hep G2 and Huh 7 cells, respectively. **g** and **h** MiRNP IP assay showing the association between miR-500a-3p and SOCS2, SOCS4 and PTPN11 transcripts in Hep G2 and Huh 7 cells. Pulldown of IgG antibody served as the negative control

By the publicly available algorithms TargetScan and miRanda, we found that several negative regulators of JAK/STAT3 signaling, including protein inhibitors of activated STAT and suppressor of cytokine signaling (SOCS) members, SOCS2, SOCS4, SOCS6, and tyrosine phosphatases members,PTPN4 and PTPN11, may be potential target of miR-500a-3p (Fig. 2a). We exogenously overexpressed miR-500a-3p via virus transduction, and endogeneously silenced miR-500a-3p by transfecting antagomir-500a-3p (Additional file 6: Figure S2A). PCR and Western blotting revealed that miR-500a-3p overexpression reduced the mRNA and protein expression levels of SOCS2, SOCS4 and PTPN11, but SOCS6 and PTPN4 were not changed. In contrast, silencing miR-500a-3p increased the expression of SOCS2, SOCS4 and PTPN11 (Fig. 2b-d). Luciferase assay revealed that upregulating miR-500a-3p decreased, while silencing miR-500a-3p increased the reporter activity driven by the 3′UTRs of SOCS2, SOCS4 and PTPN11 transcripts in dose-dependent manners, but not by the mutant 3′UTRs of these transcripts within miR-500a-3p-binding seed regions in HCC cells (Fig. 2e and f and Additional file 6: Figure S2B). Furthermore, microribonucleoprotein (miRNP) immunoprecipitation (IP) assay demonstrated a selective association of miR-500a-3p with SOCS2, SOCS4 and PTPN11 transcripts (Fig. 2g and h), further elucidating the direct repressive effect of miR-500a-3p on these regulators. Consequently, our results indicate that SOCS2, SOCS4 and PTPN11 are the direct targets of miR-500a-3p in HCC cells.

### miR-500a-3p activates STAT3 signaling pathway

**Fig. 3:**
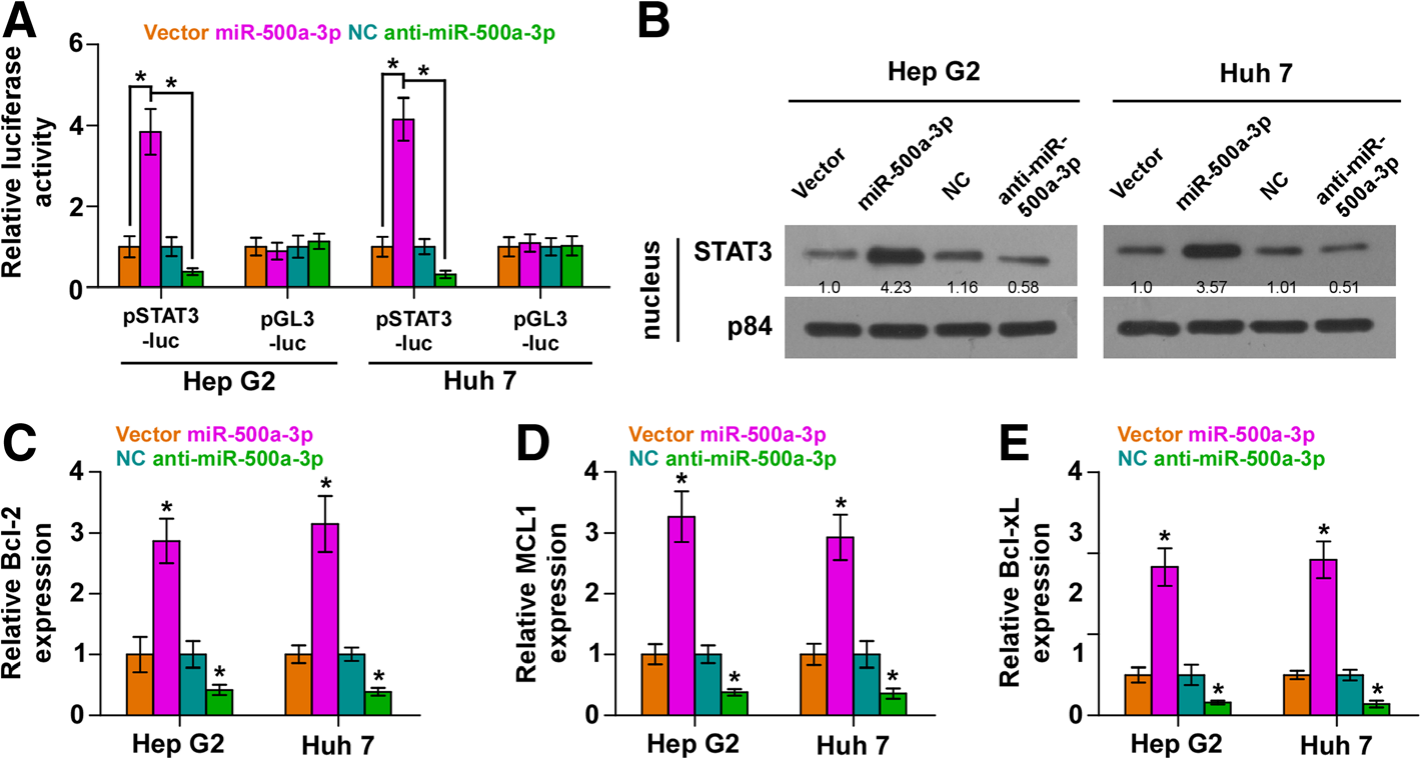
miR-500a-3p activates STAT3 signaling pathways. **a** STAT3 transcriptional activity was assessed by luciferase reporter constructs. **b** Western blotting of nuclear STAT3 expression. The nuclear protein p84 was used as the nuclear protein marker. c-**e** Real-time PCR analysis of Bcl2, MCL1 and Bcl-xL in the indicated cells

We further examined the role of miR-500a-3p in STAT3 signaling pathway in HCC. As shown in Fig. 3a, miR-500a-3p overexpression in HCC cells significantly increased, while silencing miR-500a-3p decreased, STAT3-dependent luciferase activity. Cellular fractionation and western blotting analysis revealed that upregulating miR-500a-3p increased nuclear accumulation of STAT3, while silencing miR-500a-3p reduced the nuclear translocation of STAT3 (Fig. 3b). We further examined the expression levels of multiple downstream genes of STAT3 signaling pathway including Bcl-2, MCL1 and Bcl-xL and found that overexpression of miR-500a-3p enhanced, while silencing miR-500a-3p decreased the expression of Bcl-2, MCL1 and Bcl-xL (Fig. 3c-e). Moreover, to determine whether miR-500a-3p activates STAT3 signaling in HCC cells via targeting SOCS2, SOCS4 and PTPN11, we further knocked down SOCS2, SOCS4 and PTPN11 in miR-500a-3p-silencing cells (Additional file 7: Figure S3A).As shown in Additional file 7: Figure S3B, individual silencing of SOCS2, SOCS4 and PTPN11 reversed the STAT3 activity inhibited by anti-miR-500a-3p. Thus, these results indicated that miR-500a-3p activates STAT3 signaling pathways via targeting SOCS2, SOCS4 and PTPN11 in HCC cells.

### miR-500a-3p promotes CSC-like phenotypes via targeting these negative regulators of STAT3 signaling in HCC cells

**Fig. 4:**
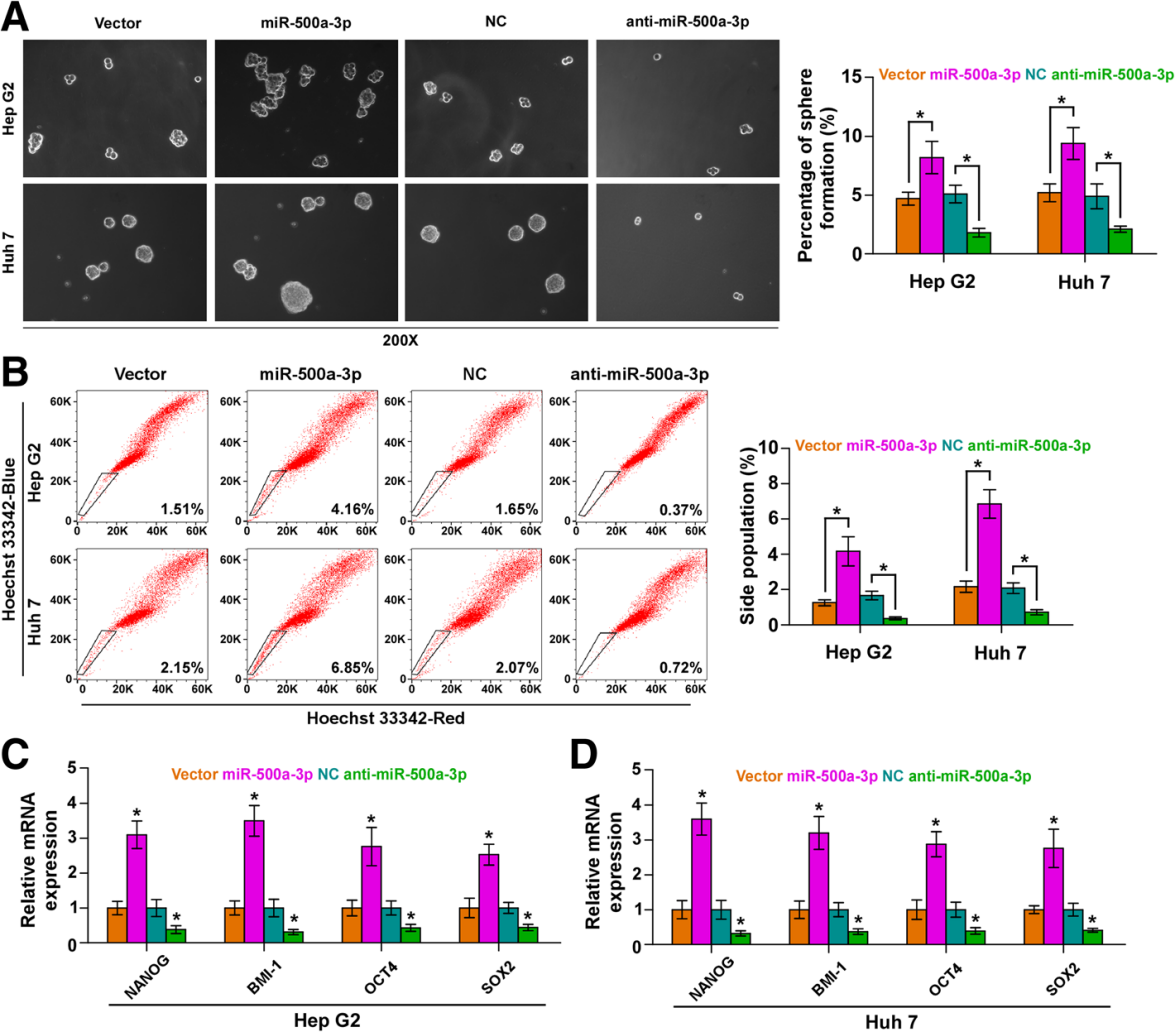
miR-500a-3p promotes stem cell properties in hepatocellular carcinoma cells. **a** Representative images of spheres formed at 200-fold magnification were counted. Histograms show the mean number of spheres formed. **b** Hoechst 33,342 dye exclusion assay showed that overexpressing miR-500a-3p promoted the fraction of side population, whereas silencing miR-500a-3p decreased the fraction. **c** and **d** Real-time PCR analysis of OCT4A, SOX2, NANOG and BMI-1 expression. GAPDH was used as the loading control. Error bars represent the mean ± SD of three independent experiments. Error bars represent the mean ± S.D. of three independent experiments. *P < 0.05

Accumulating studies reported that STAT3 signaling pathway played important roles in the stemness maintenance of cancer stem cell [6, 9]. We further investigated the effect of miR-500a-3p in cancer stem cell properties. Sphere formation assay revealed that overexpression of miR-500a-3p increased sphere formation ability in HCC cells, while silencing miR-500a-3p decreased sphere formation ability (Fig. 4a). Side population (SP) analysis was performed and the results indicated that upregulation of miR-500a-3p increased, while silencing miR-500a-3p decreased the fraction of SP cells (Fig. 4b). In addition, real-time PCR showed that miR-500a-3p upregulation significantly increased, but anti-miR-500a-3p decreased, pluripotency-associated markers, including NANOG, BMI-1, OCT4 and SOX2 (Fig. 4c and d). Furthermore, individual silencing of SOCS2, SOCS4 and PTPN11 rescued the inhibitory effects of anti-miR-500a-3p on the sphere formation ability and fraction of SP cells (Additional file 8: Figure S4A and 4B). Collectively, these results indicated that miR-500a-3p promotes CSC-like phenotypes of HCC cell via targeting SOCS2, SOCS4 and PTPN11, which further promotes the progression of hepatocellular carcinoma.

### miR-500a-3p promotes tumorigenesis of HCC cells in vivo

**Fig. 5:**
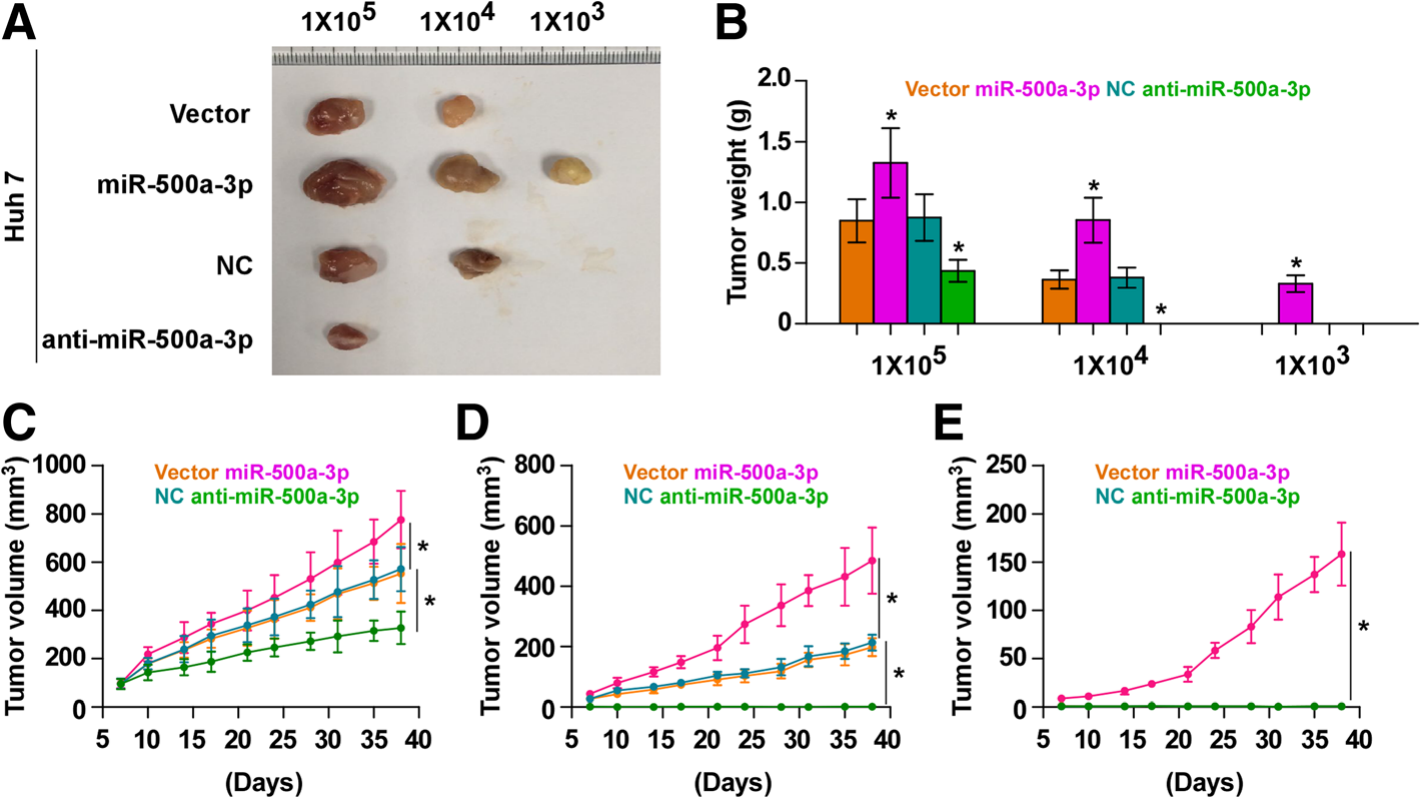
miR-500a-3p promotes the tumorigenicity of hepatocellular carcinoma cells *in vivo*. **a** Representative images of tumors in xenograft model of nude mice. Tumors formed by overexpressed miR-500a-3p Huh 7cells grew quickly than the control tumors in the 1 × 10^5^ and 1 × 10^4^ cell group. Conversely, tumors formed by anti-miR-500a-3p cells were smaller than tumors formed by NC vector cells. Only miR-500a-3p-overexpressing cells formed tumors following implantation of 1 × 10^3^ cells. **b** Histograms show the mean tumor weights of each group (*n* = 6 per group). *P < 0.05. (C-E) Growth curves for tumor formation after implantation. Mean tumor volumes are plotted

The effect of miR-500a-3p on tumorigenesis of HCC cells was further investigated in vivo. The miR-500a-3p-overexpressing, miR-500a-3p-silenced and control cells of Huh 7 were inoculated into nude mice, respectively. Tumors formed by miR-500a-3p-overexpressing cells were larger than tumors in the control group after implantation of 1 × 10^5^ or 1 × 10^4^ cells, whereas tumors in the miR-500a-3p-silencing group grew slowly than the tumors in the NC group (Fig. 5a-d). It’s worth noting that tumors were only detected in miR-500a-3p-overexpressing cells when 1 × 10^3^ cells were inoculated (Fig. 5e and Additional file 9: Figure S5). These findings indicated that miR-500a-3p promotes tumorigenesis of HCC cells in vivo.

### miR-500a-3p regulates CSC-like phenotypes via activating STAT3 signaling in HCC

**Fig. 6:**
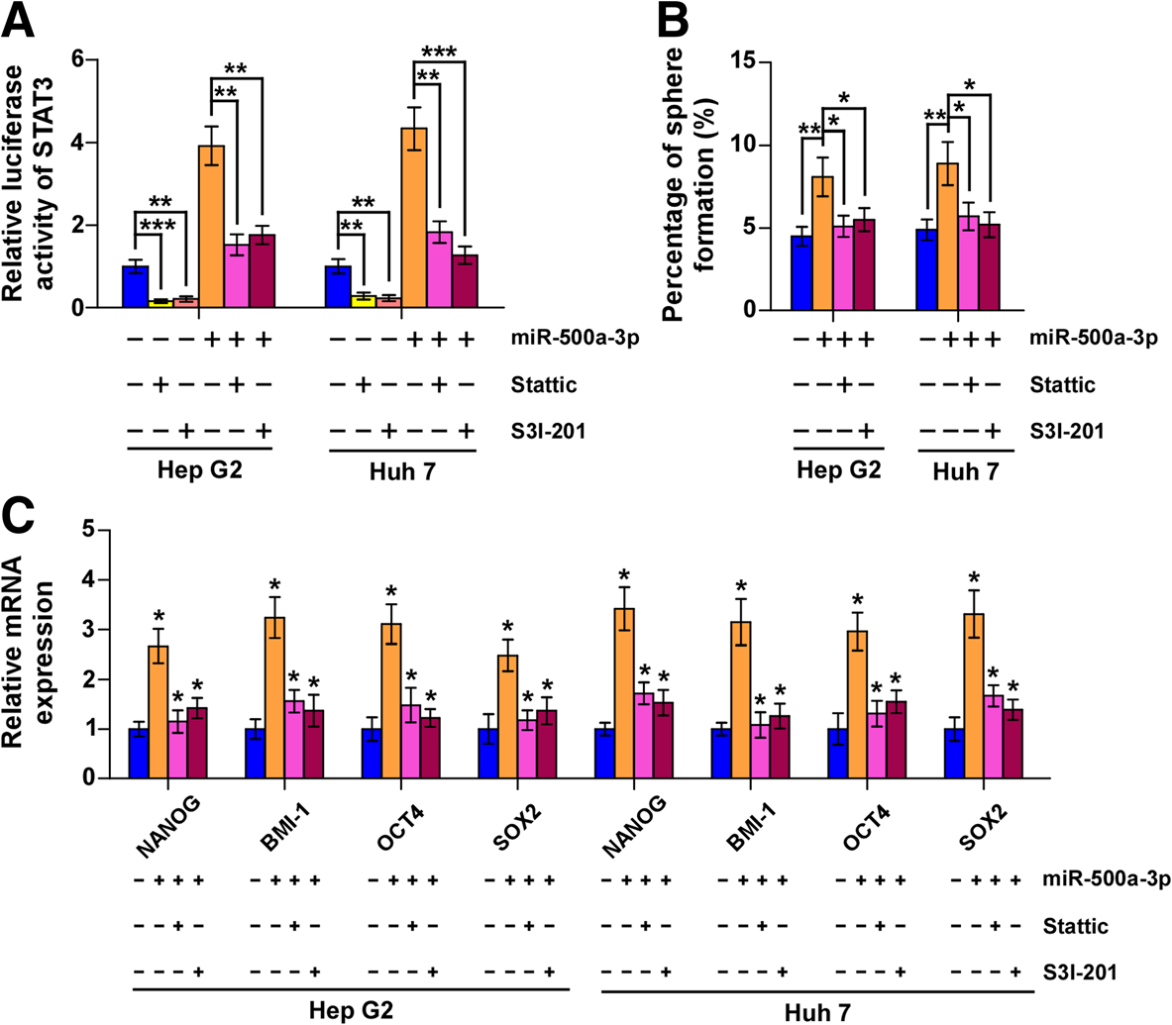
miR-500a-3p promotes stem cell properties via STAT3 signaling in hepatocellular carcinoma. **a** STAT3 inhibitors Stattic (5 μM) and S3I-201 (100 μM) showed potent inhibition of the STAT3 reporter activity in the indicated cells. Error bars represent the mean ± s.d. of three independent experiments. *P < 0.05, **P < 0.01 and ***P < 0.001. **b** Sphere formation assay showed the sphere formation ability of the indicated cells under treatment of the inhibitors Stattic and S3I-201. Error bars represent the mean ± s.d. of three independent experiments. *P < 0.05, **P < 0.01 and ***P < 0.001. **c** Real-time PCR analysis of the expression of NANOG, BMI-1, OCT4 and SOX2 under treatment of the inhibitors Stattic and S3I-201. GAPDH was used as the loading control. Error bars represent the mean ± s.d. of three independent experiments. *P < 0.05, **P < 0.01 and ***P < 0.001

We further examined the functional significance of STAT3 signaling in the stemness of HCC cells by using STAT3 signaling inhibitors. As shown in Fig. 6a, STAT3 inhibitors Stattic and S3I-201 showed potent inhibition of the STAT3 reporter activity, as well as antagonized the stimulatory effect of miR-500a-3p on STAT3 activity. The promoting effect of miR-500a-3p on sphere formation was attenuated by the STAT3 inhibitors (Fig. 6b). Moreover, inhibition of STAT3 signaling reversed the increased expression of cancer stem cell factors by miR-500a-3p overexpression (Fig. 6c). These results suggest that activation of STAT3 signaling is critical for miR-500a-3p–induced stemness in HCC cells.

### Clinical association of miR-500a-3p with SOCS2, SOCS4 and PTPN11 expression in human hepatocellular carcinoma tissues

**Fig. 7:**
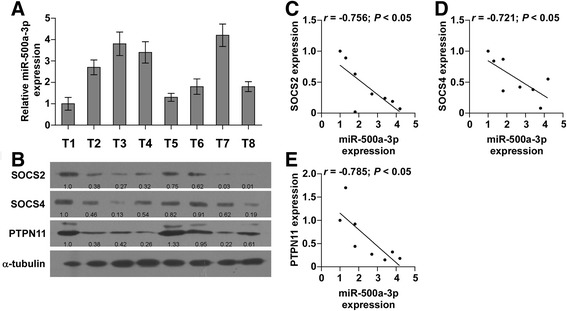
Clinical relevance of miR-500a-3p with SOCS2, SOCS4 and PTPN11 in hepatocellular carcinoma tissues. **a** Real-time PCR of miR-500a-3p expression in eight fresh hepatocellular carcinoma tissues. U6 was used as the control for RNA loading; miRNA levels were normalized to that miR-500a-3p expression of sample one. Each bar represents the mean ± SD of three independent experiments. **b** Western blotting of SOCS2, SOCS4 and PTPN11 expression in the same eight hepatocellular carcinoma tissues. α-tubulin was used as the loading control. **c**-**e** Correlation between miR-500a-3p levels and SOCS2, SOCS4 and PTPN11 expression in hepatocellular carcinoma clinical tissues. The expression levels of SOCS2, SOCS4 and PTPN11 were quantified by densitometry using Quantity One Software, and normalized to the levels of α-tubulin. The sample one was used as a standard. The relative expressions of these proteins were used to perform the correlation analysis

To assess the clinical correlation of miR-500a-3p with SOCS2, SOCS4 and PTPN11 expression, we examined the miR-500a-3p expression and protein levels of SOCS2, SOCS4 and PTPN11 in eight frozen human HCC tissues. As shown in Fig. 7a-e, miR-500a-3p expression level in HCC tissues was negatively associated with expression levels of SOCS2 (*r* = −0.756, *P* < 0.05), SOCS4 (*r* = −0.721, *P* < 0.05), and PTPN11 (*r* = −0.785, *P* < 0.05). Furthermore, miR-500a-3p levels in HCC tissues were positively associated with mRNA levels of the STAT3 downstream genes Bcl-2 (*r* = 0.725, *P* = 0.042), MCL1 (*r* = 0.719, *P* = 0.044) and Bcl-xL (*r* = 0.779, *P* = 0.029) (Additional file 10: Figure S6). Taken together, these findings demonstrated that expression level of miR-500a-3p in HCC tissues negatively correlates with SOCS2, SOCS4 and PTPN11.

## Discussion

In the present study, we report that oncogenic miR-500a-3p promotes the tumorigenesis via regulating CSC-like phenotypes in hepatocellular carcinoma. Furthermore, our results reveal that miR-500a-3p targets several negative regulators of STAT3 signaling pathway, including SOCS2, SOCS4 and PTPN11, leading to the activation of STAT3 signaling. These results determine a critical role of miR-500a-3p in the tumorigenesis of HCC.

Extensive studies have indicated that CSCs were associated with a high tumorigenicity, invasive behaviour and poor clinical outcome, driving the recurrence and distant metastasis of cancer [32, 33]. Indeed, CSCs have also been reported to play pivotal roles in hepatocellular carcinoma and indicate poor prognosis in HCC patients [34, 35]. Furthermore, therapy targeting CSCs within HCC exhibited favorable prospects in the treatment of hepatocellular carcinoma. Zhang et al. reported that a monoclonal antibody against recurrent HCC, 1B50–1, had a therapeutic effect on HCC engraftments by eliminating CSCs [36]. Despite substantial evidence for the clinical relevance of CSCs, the specific mechanisms underlying the stem maintenance of the CSC population in hepatocellular carcinoma remain largely unknown. In this study, we found that upregulating miR-500a-3p decreases the HCC cell number required to initiate tumorigenesis in vivo, indicating that overexpression of miR-500a-3p promotes the progression of HCC via enhancing CSCs characteristics. Importantly, silencing miR-500a-3p dramatically inhibits CSCs characteristics in vitro and tumorigenesis in vivo Thus, our findings not only unravel a novel mechanism for the maintenance of CSCs in hepatocellular carcinoma, but also provide a novel target for restraining the CSC population in hepatocellular carcinoma.

Numerous studies reported that STAT3 signaling played important roles in several aspects of tumorigenesis via regulating cell proliferation, apoptosis, increased resistance to chemotherapeutic agents, cancer stem cell and metastasis, leading to the development and progression of cancer [9, 37, 38]. Furthermore, STAT3 signaling has been identified to promote the progression and metastasis via inducing cancer stem cell-like properties in variety of cancers, including hepatocellular carcinoma. [38, 39]. Indeed, Terence and the colleagues reported that CD24^+^ liver tumor-initiating cells drive self-renewal and tumor initiation through STAT3-inducing NANOG expression [7], indicating that STAT3 signaling played important roles in the maintenance of CSCs in HCC. Emerging evidence indicated that deregulation of negative regulators of JAK/STAT3 signaling pathway, including tyrosine phosphatase and SOCS families contributed to the activation of JAK/STAT3 signaling [40]. For example, Banibrata reported that SOCS2 expression is necessary for the inhibition of STAT3 signaling by c-Src inhibitors. Overexpression of SOCS2 is adequate to prevent STAT3 reactivation and to enhance the cytotoxic effects of c-Src inhibition in head and neck squamous carcinoma [41]. Furthermore, PTPN11 has been identified to act as a tumor suppressor in hepatocellular carcinogenesis. Decreased PTPN11 expression was detected in a subfraction of human hepatocellular carcinoma specimens [42]. Tumbar and the colleagues reported that transcriptional repression of SOCS3 and SOCS4 by RUNX1 were essential for cancer cell growth in oral, skin, and ovarian epithelial human cancer cells [43]. However, the molecular mechanisms responsible for the activation of STAT3 signaling activation in HCC are still poorly understood. In this study, our results demonstrated that miR-500a-3p activates STAT3 signaling via directly targeting multiple negative regulators of STAT3 signaling, including SOCS2, SOCS4 and PTPN11. Thus, these findings uncover a novel mechanism responsible for the activation of STAT3 in HCC, which further promotes the progression of HCC.

Several studies reported that miR-500a was upregulated in human cancer, for example, chronic lymphocytic leukemia [44], and non-neoplastic diseases, such as endometriosis [45]. Interestingly, Zhang and the colleagues revealed that miR-500a was higher in HCC patients than in patients with chronic hepatitis B (CHB) and in healthy controls, but there was no great difference between CHB patients and healthy controls. More importantly, their findings suggest that serum miR-500a might serve as novel, noninvasive biomarkers for the diagnosis of HCC [46]. However, the specific mechanisms by which miR-500a promotes the progression of HCC remain poorly known. Consistent with these findings, we found that miR-500a-3p was upregulated in HCC tissues and cells and overexpression of miR-500a-3p correlated with poor overall and relapse-free survival in HCC patients. Furthermore, upregulation of miR-500a-3p promoted, while silencing miR-500a-3p repressed, the CSCs-like phenotypes in vitro and tumorigenicity in vivo in hepatocellular carcinoma cells via STAT3 signaling. These findings indicate that miR-500a-3p plays an important role in the development and progression of HCC.

## Conclusion

In summary, this study reports that oncogenic miR-500a-3p promotes the CSCs properties and tumourigenicity by negatively regulating SOCS2, SOCS4 and PTPN11, leading to activation of the STAT3 signalling pathway in hepatocellular carcinoma. Improved understanding the specific role of miR-500a-3p in the activation of STAT3 signaling pathway and in the pathogenesis of hepatocelluar carcinoma facilitates the development of novel therapeutic methods in the treatment of hepatocellular carcinoma.

## Additional files

Additional file 1: Table S1. A list of primers used in the reactions for clone PCR.

Additional file 2: Table S2. A list of primers used in the reactions for real-time RT-PCR.

Additional file 3: Figure S1. (A) miR-500a-3p expression level in 73 paired primary hepatocellular carcinoma tissues compared with matched adjacent normal tissues in the miRNA sequencing hepatocellular carcinoma dataset of the E-GEOD-21362. Transcript levels were normalized to U6 expression. *P* < 0.001. (B) miR-500a-3p expression level in hepatocellular carcinoma tissues in the miRNA sequencing hepatocellular carcinoma dataset from E-GEOD-31384 (Hepatocellular carcinoma, *n* = 166). (C) Kaplan–Meier analysis of overall survival curves of patients with hepatocellular carcinoma from TCGA in high miR-500a-3p expression (*n* = 191) and low miR-500a-3p expression (*n* = 181). *P* = 0.008. (D) Kaplan–Meier analysis of relapse-free survival curves of patients with hepatocellular carcinoma from TCGA in high miR-500a-3p expression (*n* = 179) and low miR-500a-3p expression (*n* = 167). *P* < 0.001.

Additional file 4: Table S3. The clinicopathological characteristics in 120 hapatocellular carcinoma patients.

Additional file 5: Table S4. The correlation between miR-500a-3p and clinicopathological characteristics in 120 patients with hepatocellular carcinoma.

Additional file 6: Figure S2. (A) Real-time PCR analysis of miR-500a-3p expression in Hep G2 and Huh 7 cells transduced with miR-500a-3p or transfected with anti-miR-500a-3p compared to control. Transcript levels were normalized by U6 expression. Error bars represent the mean ± s.d. of three independent experiments. *P < 0.05. (B) Mutant sequence in 3′UTRs of SOCS2, SOCS4, and PTPN11

Additional file 7: Figure S3. (A) Western blotting analysis of SOCS2, SOCS4 and PTPN11 expression after individual silencing of SOCS2, SOCS4 and PTPN11 respectively in miR-500a-3p silencing HCC cells. α-Tubulin served as the loading control. (B) Individual silencing of SOCS2, SOCS4 and PTPN11 rescued the STAT3 activity repression in miR-500a-3p-silencing cells. *P < 0.05, **P < 0.01 and ***P < 0.00.1.

Additional file 8: Figure S4. (A) Individual silencing of SOCS2, SOCS4 and PTPN11 rescued the sphere formation repressed by anti-miR-500a-3p. *P < 0.05, **P < 0.01 and ***P < 0.00.1. (B) Individual silencing of SOCS2, SOCS4 and PTPN11 rescued the fraction of SP cells repressed by anti-miR-500a-3p. *P < 0.05, **P < 0.01 and ***P < 0.00.1.

Additional file 9: Figure S5. The number of tumor formation initiated by different amounts of Huh 7 cells in nude mice.

Additional file 10: Figure S6. Clinical relevance of miR-500a-3p with the expression of downstream genes of STAT3 signaling in hepatocellular carcinoma. (A-C) Correlation analysis of miR-500a-3p expression and Bcl-2, Bcl-xL and MCL1 mRNA expression levels in 8 fresh human hepatocellular carcinoma tissues.

## Abbreviations

ABCG2: ATP-binding cassette, sub-family G (WHITE) member 2
BMI1: Proto-oncogene polycomb ring finger
CSCs: Cancer stem cell
HCC: Hepatocellular carcinoma
JAK/STAT: Janus kinase/signal transducer and activator of transcription
NANOG: Nanog homeobox
OCT4A: POU class 5 homeobox 1A
PTPN: Protein tyrosine phosphatase
SOCS: Suppressor of cytokine signaling
