# Supplementary material for: miR-500a-3p promotes cancer stem cells properties via STAT3 pathway in human hepatocellular carcinoma
Source: J Exp Clin Cancer Res. 2017 Jul 27;36:99. doi: 10.1186/s13046-017-0568-3 (PMC5532790; doi:10.1186/s13046-017-0568-3)
Supplement: Supplementary file 1 — A list of primers used in the reactions for clone PCR. [file 13046_2017_568_MOESM1_ESM.pdf]

Table S1. A list of primers used in the reactions for clone PCR.

**Used for subcloning and plasmid construction:**	
miR-500a-clone-F	TTGAACCAAGGTTCGTAAATACCAA
miR-500a-clone-R	AAAGGCAGAGAATCTTTTCTCCTGC
SOCS2-3UTR-22nt-clone-F	TGTCTCACATAGAGTATCTCCGAAT
SOCS2-3UTR-1566nt-clone-R	TCCAGTAAGCAGGTTTTATTACAAT
SOCS4-3UTR-44nt-clone-F	TAACAGGATGGGAACATGGG
SOCS4-3UTR-1585nt-clone-R	CTTACCACACTATTTTCTTTCACCA
PTPN11-3UTR-198nt-clone-F	CTTCCCAATTACTCATTTCCTCA
PTPN11-3UTR-1993nt-clone-R	CCAAACTACCCCAAAGTCTCAA
