# Supplementary material for: miR-500a-3p promotes cancer stem cells properties via STAT3 pathway in human hepatocellular carcinoma
Source: J Exp Clin Cancer Res. 2017 Jul 27;36:99. doi: 10.1186/s13046-017-0568-3 (PMC5532790; doi:10.1186/s13046-017-0568-3)
Supplement: Supplementary file 2 — A list of primers used in the reactions for real-time RT-PCR. [file 13046_2017_568_MOESM2_ESM.pdf]

TableS2. A list of primers used in the reactions for real-time RT-PCR.

**Real-time PCR primer:**	
PTPN4-up	CAACAAGACTTGCCCCTGAT
PTPN4-dn	GCCCGAGACAGACAGCATAC
PTPN11-up	CTGCCTCCACACCAGTGATA
PTPN11-dn	GGAGCCTGAGCAAGGAGC
SOCS2-up	GGAGGACGGATGACAAAGTC
SOCS2-dn	AGACACTCTCCGGACTGAGG
SOCS4-up	TTCTTCTGGGCACTTTCTGG
SOCS4-dn	AGACTGATGGCGATGGTGAT
SOCS6-up	CTTCTGTGCCAGTGAGTCCA
SOCS6-dn	ACGATGATGTCCCTCCACTC
BCL-2-up	GTGGATGACTGAGTACCTGAACC
BCL-2-dn	AGACAGCCAGGAGAAATCAAAC
MCL1-up	GAGGAGGACGAGTTGTACCG
MCL1dn	ATGTCCAGTTTCCGAAGCAT
BCL-xL-up	GGTATTGGTGAGTCGGATCG
BCL-xL-dn	TGCTGCATTGTTCCCATAGA
GAPDH-up	ATTCCACCCATGGCAAATTC
GAPDH-dn	TGGGATTTCCATTGATGACAAG
