# Supplementary figures and images for: miR-500a-3p promotes cancer stem cells properties via STAT3 pathway in human hepatocellular carcinoma
Source: J Exp Clin Cancer Res. 2017 Jul 27;36:99. doi: 10.1186/s13046-017-0568-3 (PMC5532790; doi:10.1186/s13046-017-0568-3)

Supplemental Figure 1

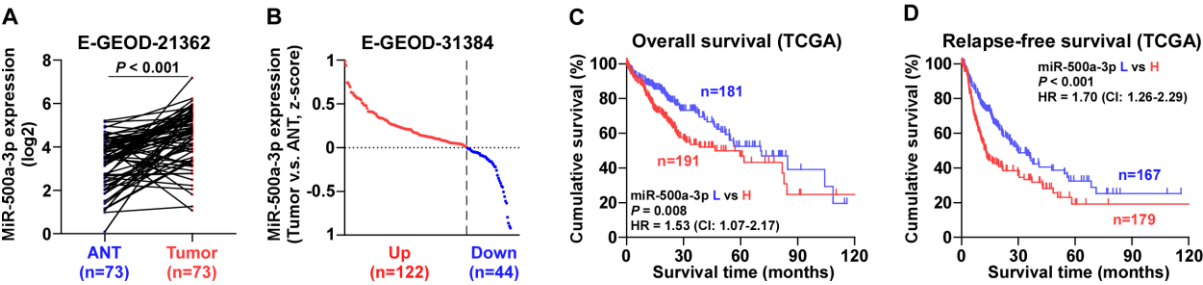

Supplement: Supplementary file 3 — (A) miR-500a-3p expression level in 73 paired primary hepatocellular carcinoma tissues compared with matched adjacent normal tissues in the miRNA sequencing hepatocellular carcinoma dataset of the E-GEOD-21362. Transcript levels were normalized to U6 expression. P < 0.001. (B) miR-500a-3p expression level in hepatocellular carcinoma tissues in the miRNA sequencing hepatocellular carcinoma dataset from E-GEOD-31384 (Hepatocellular carcinoma, n = 166). (C) Kaplan–Meier analysis of overall survival curves of patients with hepatocellular carcinoma from TCGA in high miR-500a-3p expression (n = 191) and low miR-500a-3p expression (n = 181). P = 0.008. (D) Kaplan–Meier analysis of relapse-free survival curves of patients with hepatocellular carcinoma from TCGA in high miR-500a-3p expression (n = 179) and low miR-500a-3p expression (n = 167). P < 0.001. [file 13046_2017_568_MOESM3_ESM.pdf]

Supplemental Figure 2

A

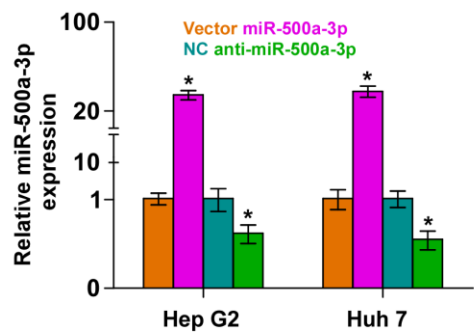

B

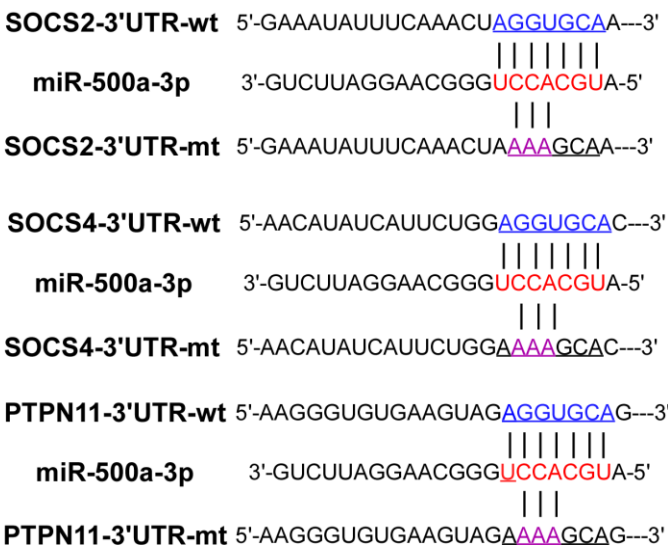

Supplement: Supplementary file 6 — (A) Real-time PCR analysis of miR-500a-3p expression in Hep G2 and Huh 7 cells transduced with miR-500a-3p or transfected with anti-miR-500a-3p compared to control. Transcript levels were normalized by U6 expression. Error bars represent the mean ± s.d. of three independent experiments. *P < 0.05. (B) Mutant sequence in 3′UTRs of SOCS2, SOCS4, and PTPN11 [file 13046_2017_568_MOESM6_ESM.pdf]

Supplemental Figure 3

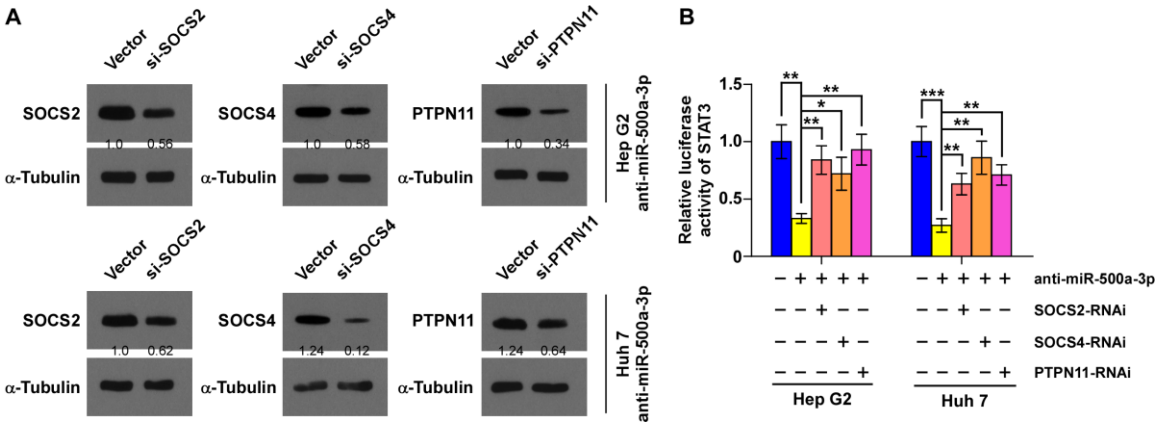

Supplement: Supplementary file 7 — (A) Western blotting analysis of SOCS2, SOCS4 and PTPN11 expression after individual silencing of SOCS2, SOCS4 and PTPN11 respectively in miR-500a-3p silencing HCC cells. α-Tubulin served as the loading control. (B) Individual silencing of SOCS2, SOCS4 and PTPN11 rescued the STAT3 activity repression in miR-500a-3p-silencing cells. *P < 0.05, **P < 0.01 and ***P < 0.00.1. [file 13046_2017_568_MOESM7_ESM.pdf]

Supplemental Figure 4

A

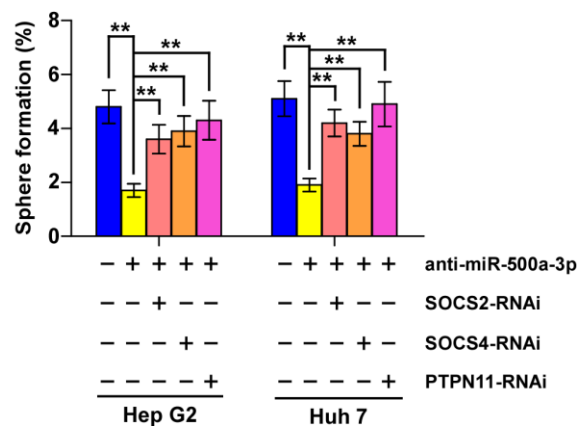

B

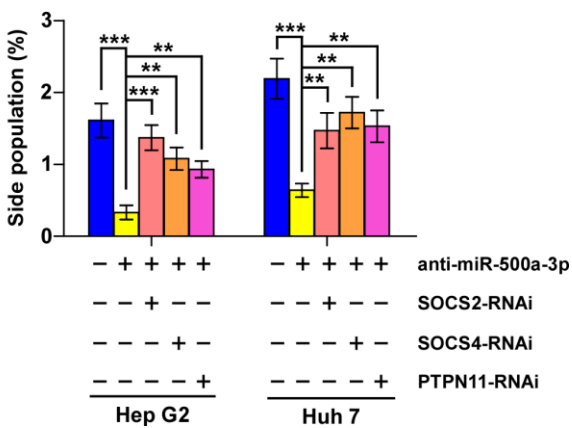

Supplement: Supplementary file 8 — (A) Individual silencing of SOCS2, SOCS4 and PTPN11 rescued the sphere formation repressed by anti-miR-500a-3p. *P < 0.05, **P < 0.01 and ***P < 0.00.1. (B) Individual silencing of SOCS2, SOCS4 and PTPN11 rescued the fraction of SP cells repressed by anti-miR-500a-3p. *P < 0.05, **P < 0.01 and ***P < 0.00.1. [file 13046_2017_568_MOESM8_ESM.pdf]

Supplemental Figure 6

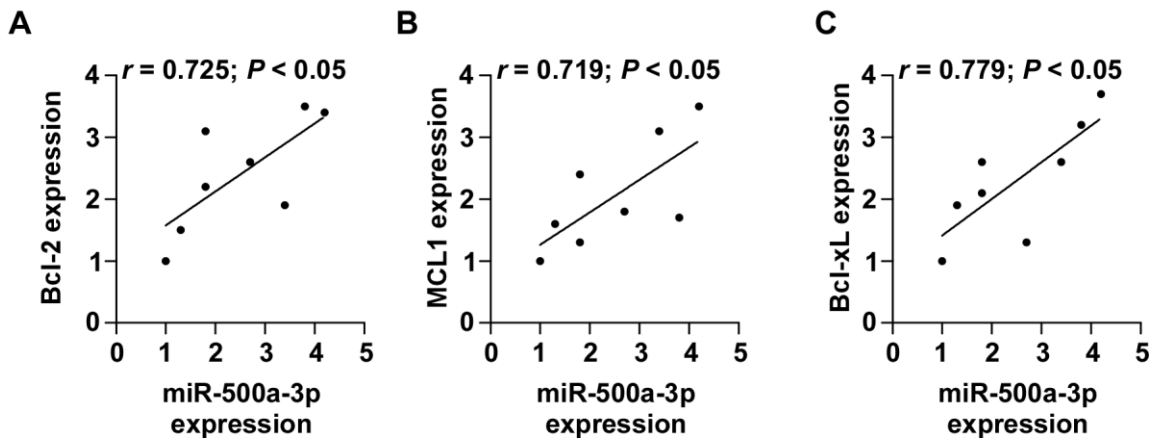

Supplement: Supplementary file 10 — Clinical relevance of miR-500a-3p with the expression of downstream genes of STAT3 signaling in hepatocellular carcinoma. (A-C) Correlation analysis of miR-500a-3p expression and Bcl-2, Bcl-xL and MCL1 mRNA expression levels in 8 fresh human hepatocellular carcinoma tissues. [file 13046_2017_568_MOESM10_ESM.pdf]
