# Supplementary material for: miR-500a-3p promotes cancer stem cells properties via STAT3 pathway in human hepatocellular carcinoma
Source: J Exp Clin Cancer Res. 2017 Jul 27;36:99. doi: 10.1186/s13046-017-0568-3 (PMC5532790; doi:10.1186/s13046-017-0568-3)
Supplement: Supplementary file 4 — The clinicopathological characteristics in 120 hapatocellular carcinoma patients. [file 13046_2017_568_MOESM4_ESM.pdf]

Table S3. The clinicopathological characteristics in 120 hapatocellular carcinoma patients

Parameters	Number of cases	Parameters	Number of cases
Gender		Age (years)	
Female	42	<60	57
Male	78	≥60	63
AFP		Differentiation	
<400	50	High/moderate	50
≥400	70	Poor	70
Clinical stage		T stage	
I	28	$T_1 – T_2$	62
II – IV	92	$T_3 – T_4$	58
N stage		M stage	
N_0	49	M_0	57
N_1	71	M_1	63
Survival status		Tumor size (5 cm)	
Alive	67	<5	50
Dead	53	≥5	70
Venous invasion			
Negative	58		
Positive	62		

Abbreviation: AFP, alpha fetoprotein.
