# Supplementary material for: miR-500a-3p promotes cancer stem cells properties via STAT3 pathway in human hepatocellular carcinoma
Source: J Exp Clin Cancer Res. 2017 Jul 27;36:99. doi: 10.1186/s13046-017-0568-3 (PMC5532790; doi:10.1186/s13046-017-0568-3)
Supplement: Supplementary file 5 — The correlation between miR-500a-3p and clinicopathological characteristics in 120 patients with hepatocellular carcinoma. [file 13046_2017_568_MOESM5_ESM.pdf]

Table S4. The correlation between miR-500a-3p and clinicopathological characteristics in 120 patients with hepatocellular carcinoma.

Parameters	Number of cases	miR-500a-3p expression		*P* values
		Low	High	
Gender				
Female	42	24	18	0.251
Male	78	36	42	
Age (years)				
<60	57	32	25	0.200
≥60	63	28	35	
AFP				
<400	50	31	19	0.026*
≥400	70	29	41	
Differentiation				
High/moderate	50	27	23	0.459
Poor	70	33	37	
T stage				
$T_1 – T_2$	62	34	28	0.273
$T_3 – T_4$	58	26	32	
N stage				
N_0	49	28	21	0.194
N_1	71	32	39	
M stage				
M_0	57	36	21	0.006*
M_1	63	24	39	
Clinical stage				
I	28	20	8	0.010*
II - IV	92	40	52	
Tumor size (cm)				
<5	50	31	19	0.041*
≥5	70	29	41	
Venous invasion				
Negative	58	35	23	0.029*
Positive	62	25	37	
