# Supplementary material for: miR-500a-3p promotes cancer stem cells properties via STAT3 pathway in human hepatocellular carcinoma
Source: J Exp Clin Cancer Res. 2017 Jul 27;36:99. doi: 10.1186/s13046-017-0568-3 (PMC5532790; doi:10.1186/s13046-017-0568-3)
Supplement: Supplementary file 9 — The number of tumor formation initiated by different amounts of Huh 7 cells in nude mice. [file 13046_2017_568_MOESM9_ESM.pdf]

Supplemental Figure 5

	Inoculation of Huh 7 cell number		
	$1X10^5$	$1X10^4$	$1X10^3$
control	6/6	4/6	0/6
miR-500a-3p	6/6	6/6	5/6
NC	6/6	3/6	0/6
anti-miR-500a-3p	5/6	0/6	0/6
